# Comparative Analysis of Gelsemine and *Gelsemium sempervirens* Activity on Neurosteroid Allopregnanolone Formation in the Spinal Cord and Limbic System

**DOI:** 10.1093/ecam/nep083

**Published:** 2011-06-16

**Authors:** Christine Venard, Naoual Boujedaini, Ayikoe Guy Mensah-Nyagan, Christine Patte-Mensah

**Affiliations:** ^1^Equipe “Stéroïdes, Neuromodulateurs et Neuropathologies”, EA-4438, Université de Strasbourg, Bâtiment 3 de la Faculté de Médecine, F-67000 Strasbourg, France; ^2^Laboratoires BOIRON, Sainte-Foy-lès-Lyon, France

## Abstract

Centesimal dilutions (5, 9 and 15 cH) of *Gelsemium sempervirens* are claimed to be capable of exerting anxiolytic and analgesic effects. However, basic results supporting this assertion are rare, and the mechanism of action of *G. sempervirens* is completely unknown. To clarify the point, we performed a comparative analysis of the effects of dilutions 5, 9 and 15 cH of *G. sempervirens* or gelsemine (the major active principle of *G. sempervirens*) on allopregnanolone (3**α**,5**α**-THP) production in the rat limbic system (hippocampus and amygdala or H-A) and spinal cord (SC). Indeed, H-A and SC are two pivotal structures controlling, respectively, anxiety and pain that are also modulated by the neurosteroid 3**α**,5**α**-THP. At the dilution 5 cH, both *G. sempervirens* and gelsemine stimulated [^3^H]progesterone conversion into [^3^H]3**α**,5**α**-THP by H-A and SC slices, and the stimulatory effect was fully (100%) reproducible in all assays. The dilution 9 cH of *G. sempervirens* or gelsemine also stimulated 3**α**,5**α**-THP formation in H-A and SC but the reproducibility rate decreased to 75%. At 15 cH of *G. sempervirens* or gelsemine, no effect was observed on 3**α**,5**α**-THP neosynthesis in H-A and SC slices. The stimulatory action of *G*. s*empervirens* and gelsemine (5 cH) on 3**α**,5**α**-THP production was blocked by strychnine, the selective antagonist of glycine receptors. Altogether, these results, which constitute the first basic demonstration of cellular effects of *G. sempervirens*, also offer interesting possibilities for the improvement of *G. sempervirens*-based therapeutic strategies.

## 1. Introduction

Neurons and glial cells are capable of synthesizing various bioactive steroids also called neurosteroids, which can regulate the nervous system activity via autocrine or paracrine mechanisms [1–4]. Pharmacological and behavioral studies have suggested that neurosteroids are involved in the regulation of important neurobiological mechanisms [1, 5–7]. In particular, the neurosteroid 3*α*,5*α*-tetrahydroprogesterone (3*α*,5*α*-THP), also named allopregnanolone, plays a key role in the modulation of neurological and psychopathologic symptoms such as depression, anxiety, analgesia and neurodegeneration [8–13]. 3*α*,5*α*-THP is a potent activator of the central inhibitory transmission, which acts through allosteric sites located on *γ*-amino butyric acid type A (GABA_A_) receptor [10, 14] or on strychnine-sensitive glycine receptor (Gly-R) [15, 16]. Because 3*α*,5*α*-THP endogenously synthesized in the central nervous system significantly modulates anxiety or nociceptive mechanisms through paracrine and autocrine modes [17–22], substances which are capable of stimulating 3*α*,5*α*-THP formation in neural networks appear as potentially interesting for the development of effective anxiolytic or analgesic therapies [23–30]. However, to be therapeutically effective, the candidate substances must be devoid of side effects such as nausea, vomiting tolerance, dependence or breathing failure induced by certain anxiolytics and analgesics [31–34].

For several years, preparations from the yellow jasmine or *Gelsemium sempervirens* Loganacea have been claimed to be anxiolytic and analgesic medicines but, surprisingly, scientific results from basic research supporting this assertion are extremely rare. Indeed, except two studies which showed that *G. sempervirens* preparations may prevent stress or development of spontaneous seizures *in vivo* [35, 36], there are no fundamental evidence demonstrating that *G. sempervirens* may control neurophysiological processes such anxiety and pain. In particular, the cellular and pharmacological mechanisms of action of *G. sempervirens* are completely unknown. In our endeavor to clarify this situation, we have recently investigated the cellular effects of gelsemine, the major active principle in *G. sempervirens* composition, and we observed that gelsemine stimulated dose-dependently 3*α*,5*α*-THP secretion in the rat spinal cord (SC) through activation of Gly-R [37]. To gain more insights into the cellular and pharmacological mechanisms of action of *G. sempervirens* itself, we have now combined pulse-chase experiments, high-performance liquid chromatography (HPLC) and flow scintillation detection [19, 22, 38–42] to perform a comparative analysis of the effects of *G. sempervirens* and gelsemine preparations on [^3^H]progesterone conversion into [^3^H]3*α*,5*α*-THP in the rat limbic system (hippocampus and amygdala or H-A) and SC. Indeed, the limbic system and SC are well-known for their crucial roles in the modulation of anxiety and pain, respectively [43–49]. We have also characterized pharmacologically, the main receptor involved in the mediation of *G. sempervirens* cellular effects in the limbic system and SC.

## 2. Methods

### 2.1. Animals

Adult male Sprague-Dawley rats weighing 300–350 g were used in this study. Animal care and manipulations were performed according to the European Community Council Directives (86/609/EC) and under the supervision of authorized investigators. All experiments were performed minimizing the number of animals used and their suffering in accordance with the Alsace Department of Veterinary Public Health Guide for the Care and Use of Laboratory Animals (Agreement number: 67-186). The animals were obtained from a commercial source (Janvier, France) and housed under standard laboratory conditions in a 12-h light/dark cycle with food and water *ad libitum*.

### 2.2. Chemicals and Reagents

Dichloromethane (DCM), propylene glycol and strychnine hydrochloride were purchased from Sigma-Aldrich (St. Louis, USA). Hexane and isopropanol were obtained from Fischer Bioblock Scientific (Illkirch, France). The synthesis product gelsemine came from Extrasynthese (Genay, France). Sodium chloride (NaCl) was purchased from VWR Prolabo (Fontenay-sous-Bois, France). Synthetic steroids including PROG, 5*α*-dihydroprogesterone (5*α*-DHP) and 3*α*,5*α*-THP were obtained from Steraloids (Newport, USA). Tritiated steroids such as 1,2,6,7-^3^H(N)-progesterone ([^3^H]PROG) and 9,11,12-^3^H(N)-3*α*,5*α*-tetrahydroprogesterone ([^3^H]3*α*,5*α*-THP) were supplied by PerkinElmer (Boston, USA).

### 2.3. Preparation of Gelsemine and *G. sempervirens* Dilutions

By using a conventional dilution process (pharmacological dilutions from a stock solution of 1 M of gelsemine), we have recently observed that gelsemine at 10^−10^ M stimulated 3*α*,5*α*-THP production in the rat SC [37]. Therefore, we decided to check whether or not gelsemine preparations obtained by homeopathic procedure (dilutions/dynamizations) may conserve the ability to stimulate 3*α*,5*α*-THP formation. Then, we asked Boiron Laboratories to prepare homeopathic dilutions of gelsemine starting from a hydroalcoholic solution (30% ethanol, v/v) of synthetic gelsemine (purchased from Extrasynthese, Genay France) at 1 M. Homeopathic dilutions/dynamizations were performed in cascade with de-ionized water. Consequently, the homeopathic dilutions 5, 9 and 15 cH led theoretically to gelsemine solution at 10^−10^, 10^−18^ and 10^−30^ M, respectively.

*Gelsemium sempervirens* preparations were obtained using the same homeopathic procedure but the mother tincture was a hydroalcoholic (30% ethanol, v/v) extract of *G. sempervirens* plant roots. Gelsemine quantity in *G. sempervirens* mother tincture was determined by using a quantitative HPLC method [50, 51]. Briefly, 20 *μ*l of *G. sempervirens* mother tincture was analyzed on the HPLC column, using a butylamine/water/methanol (0.1 : 22 : 78 v/v/v) gradient. Synthetic gelsemine (20 *μ*l), used as reference standard, was chromatographed under the same conditions and its elution position as well as the retention time of gelsemine present in *G. sempervirens* solution were determined by ultraviolet absorption at 255 nm. The amount of gelsemine contained in *G. sempervirens* was calculated considering areas of the peaks corresponding to synthetic gelsemine and gelsemine eluted from *G. sempervirens* solution. The concentration of gelsemine estimated from the analyses of different samples of *G. sempervirens* mother tinctures varied between 5 × 10^−3^ and 5 × 10^−4^ M.

After each dilution step, all gelsemine or *G. sempervirens* solutions were agitated at high speed. Control solutions were prepared according to the same procedure described above, using only the hydroalcoholic solution (30% ethanol, v/v), which was submitted to the dilution/dynamization cascade with de-ionized water. All gelsemine and *G. sempervirens* preparations as well as control solutions were kept at 4°C before use. Because the dilutions 5, 9 and 15 cH of *G. sempervirens* are often used for homeopathic treatments in humans [52], we decided in agreement with the company Boiron to focus our efforts on a detailed comparative analysis of the effects of these three dilutions of gelsemine and *G. sempervirens* on allopregnanolone production in H-A and SC.

### 2.4. Pulse-Chase Experiments

For each experiment, 200 mg SC (lumbar segment) or 15 mg H-A slices were preincubated for 15 min in 1.5 ml of 0.9% NaCl at 37°C. The SC and H-A were dissected in rats after deep anesthesia. The SC was removed by hydraulic extrusion and slices were made in the lumbar region. SC and H-A slices were incubated at 37°C for 3 h with 1.5 ml of 0.9% NaCl (pH 7.4) containing 50 nM [^3^H]PROG supplemented with 1% propylene glycol in the presence of tested compounds. The incubation was carried out in a water-saturated atmosphere (95% air, 5% CO_2_), which made it possible to maintain the pH at 7.4. At the end of the incubation period, the reaction was stopped by adding 500 *μ*l of ice-cold 0.9% NaCl and transferring the incubation medium in tubes into a cold water bath (0°C). Newly-synthesized neurosteroids released in the incubation medium were extracted three times with 2 ml of DCM, and the organic phase was evaporated on ice under a stream of nitrogen. The dry extracts were redissolved in 2 ml of hexane and prepurified on Sep-Pak C_18_ cartridges (Waters Associates, Milford, USA). Steroids were eluted with a solution made of 50% isopropanol and 50% hexane. The solvent was evaporated in a RC-10-10 Speed Vac Concentrator and the dry extracts were kept at −20°C until HPLC analysis. The extraction efficiency was 89 ± 7%.

### 2.5. HPLC-Flo/One Characterization of Steroids

The newly synthesized steroids extracted from the incubation medium already purified on Sep-Pak cartridges were characterized using a previously validated method which combines HPLC analysis and flow scintillation detection [19, 22, 38–42]. Briefly, the prepurified extracts were analyzed by reversed-phase HPLC on a liquid chromatograph (322 pump, UV/VIS 156 detector, Unipoint system; Gilson, Middleton, USA) equipped with a 4.6 × 250 mm SymetryShield C_18_ column (Waters Associates, Milford, USA) equilibrated with 100% hexane. The radioactive steroids were eluted at a flow rate of 0.5 ml   min ^−1^ using a gradient of isopropanol (0%–60% over 65 min) including five isocratic steps at 0% (0–10 min), 1% (30–35 min), 2% (40–45 min), 30% (50–55 min) and 60% (60–65 min). The tritiated steroids eluted from the HPLC column were directly quantified with a flow scintillation analyzer (Radiomatic Flo/One-Beta A 500; Packard Instruments, Meriden, USA) equipped with a Pentium IV PC computer (Dell-Computer-France, Lyon, France) for measurement of the percentage of total radioactivity contained in each peak. Synthetic steroids used as reference standards were chromatographed under the same conditions as the extracts obtained from the incubation media and their elution positions were determined by ultraviolet absorption using a UV/VIS 156 detector (Gilson, Middleton, USA). The elution positions of steroids change on analytic columns after the purification of a certain number of tissue extracts. Therefore, to optimize the characterization of newly synthesized neurosteroids, synthetic tritiated neuroactive steroids including [^3^H]PROG and [^3^H]3*α*,5*α*-THP were also used as reference standards, chromatographed under the same conditions as the extracts and identified by their elution times with the Flo/One computer system before and after each extract analytic run.

### 2.6. Quantification of 3*α*,5*α*-THP Released by the SC and H-A Slices and Statistical Analysis

The amount of [^3^H]3*α*,5*α*-THP newly synthesized from [^3^H]PROG and released by H-A or SC slices in the incubation medium was expressed as a percentage of the total radioactivity contained in all peaks resolved by the HPLC-Flo/One system (after analysis of the incubation medium extracts), including [^3^H]PROG itself. Each value presented is the mean (±SEM) of four independent experiments. Statistical analysis was performed with the 3.05 version of GraphPad Instat (GraphPad Software Inc., San Diego, CA, USA). The statistical significance of differences between two groups was assessed with Student's *t*-test. Analysis of variance (ANOVA) followed by Bonferroni's test was applied for multi-parameter analyses. A *P*-value of less than 0.05 was considered significant.

## 3. Results

### 3.1. Effects of Gelsemine and *G. sempervirens* Preparations on 3*α*,5*α*-THP Production in the SC and H-A

**Figure 1:**
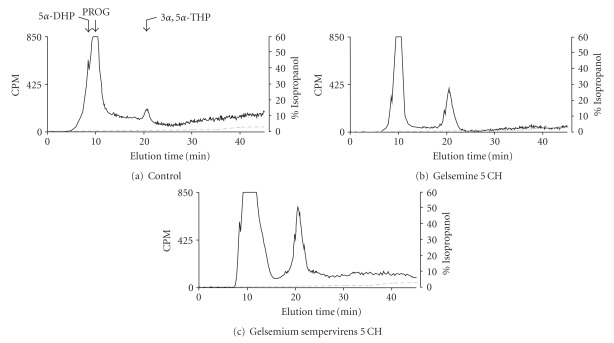
Characterization of [^3^H]-neurosteroids released in the incubation medium by: (a) lumbar SC slices after a 3-h incubation with [^3^H]PROG in the absence (b) or in presence of gelsemine 5 cH or (c) *Gelsemium sempervirens* 5 cH. Analyses were performed using a hexane/isopropanol gradient and a reverse-HPLC system coupled to a flow scintillation detector. The ordinate indicates the radioactivity measured in the HPLC eluent. The dashed line represents the gradient of secondary solvent (% isopropanol). The arrows indicate elution positions of standard steroids.

**Figure 2:**
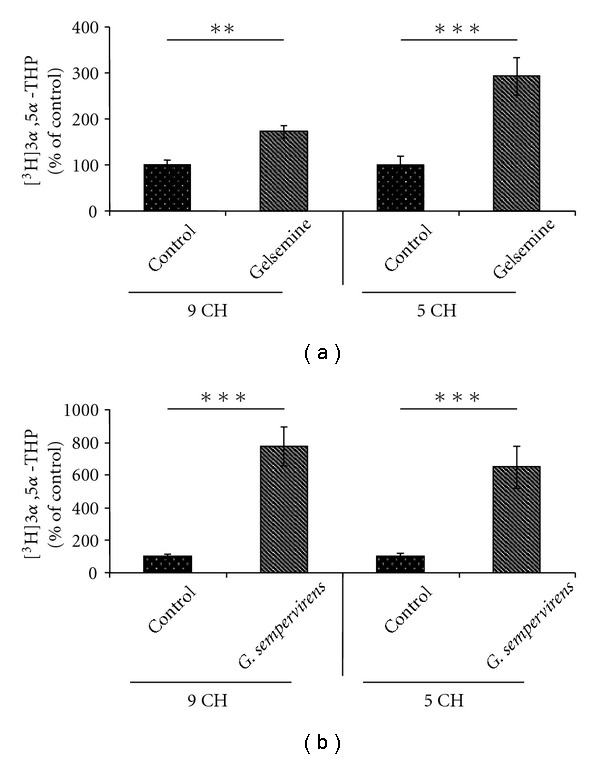
Effects of gelsemine (a) and *G. sempervirens* (b) at 9 or 5 cH on 3*α*,5*α*-THP production by SC slices. Each value was calculated as the relative amount of [^3^H]3*α*,5*α*-THP compared with the total [^3^H]-labeled compounds resolved by HPLC-Flo/One characterization (×100). The values were obtained from experiments similar to those presented in Figures 2(a). Results were then expressed as percentages of the amount of [^3^H]3*α*,5*α*-THP formed in absence of gelsemine or *G. sempervirens* (control). Each value is the mean (± SEM) of four independent experiments. **P < .01, ***P < .001 as compared to control (Student's *t*-test).

A 3-h incubation of lumbar SC slices with [^3^H]PROG yielded the formation of various radioactive metabolites (Figures 1(a)–1(c)). Reversed-phase HPLC analysis coupled with flow scintillation detection made it possible to resolve two major radioactive metabolites coeluting with [^3^H]5*α*-dihydroprogesterone ([^3^H]5*α*-DHP) and [^3^H]3*α*,5*α*-THP (Figures 1(a)–1(c)). The conversion of [^3^H]PROG into [^3^H]5*α*-DHP and [^3^H]3*α*,5*α*-THP was also observed in H-A (limbic system) slices with the same chromatographic profile as this shown in Figure 1. Investigations of the effects of gelsemine and *G. sempervirens* on [^3^H]3*α*,5*α*-THP neosynthesis in SC and H-A slices were performed by testing the dilutions 5, 9 and 15 cH. At the dilution 5 cH, gelsemine and *G. sempervirens* preparations were able to stimulate significantly [^3^H]progesterone conversion into [^3^H]3*α*,5*α*-THP in SC (Figure 2) and H-A (Figure 3). The stimulatory effect of geslmine or *G. sempervirens* at 5 cH was fully (100%) reproducible since the same effect was observed in all intra- and inter-assays performed. At the dilution 9 cH, a stimulatory action of gelsemine or *G. sempervirens* was detected on 3*α*,5*α*-THP production in SC and H-A slices (Figures 2 and 3] but this effect was observed only in 75% of the total number of samples in intra- and inter-assays investigations. No effect was detected when gelsemine and *G. sempervirens* preparations were applied at the dilution 15 cH. In the SC, *G. sempervirens* or gelsemine at 5 cH induced, respectively, a 547% or 193% increase of [^3^H]3*α*,5*α*-THP neosynthesis from [^3^H]progesterone (Figures 2(a) and 2(b)). In the limbic system or H-A, *G. sempervirens* or gelsemine at 5 cH generated, respectively, a 178% or 94% enhancement of [^3^H]3*α*,5*α*-THP formation (Figures 2(a) and 2(b)).

### 3.2. Comparative Analysis of the Stimulatory Capacity of Gelsemine and *G. sempervirens* on 3*α*,5*α*-THP Biosynthesis

**Figure 4:**
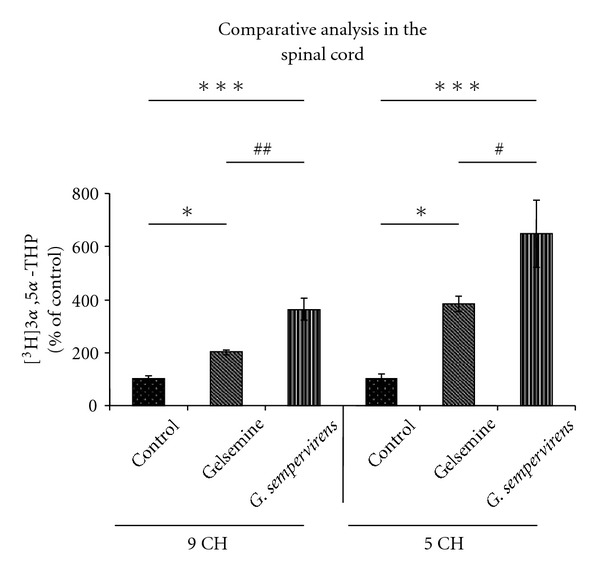
Comparative analysis of the effects of gelsemine and *G. sempervirens* at 9 or 5 cH on [^3^H]PROG conversion into [^3^H]3*α*,5*α*-THP by SC slices. The values were obtained from experiments similar to those presented in Figure 1. Results were then expressed as percentages of the amount of [^3^H]3*α*,5*α*-THP formed in the control group. Each value is the mean (±SEM) of four independent experiments. *P < .05, ***P < .001 as compared to control; ^#^ *P* < .05, ^##^ *P* < .01 as compared to gelsemine (ANOVA followed by Bonferroni's test).

In the SC, *G. sempervirens* preparations produced a stronger stimulation of 3*α*,5*α*-THP formation than gelsemine. Indeed, the level of [^3^H]3*α*,5*α*-THP newly synthesized in the presence of *G. sempervirens* at 5 cH was 1.7-fold higher than in the presence of gelsemine at 5 cH (Figure 4).

**Figure 5:**
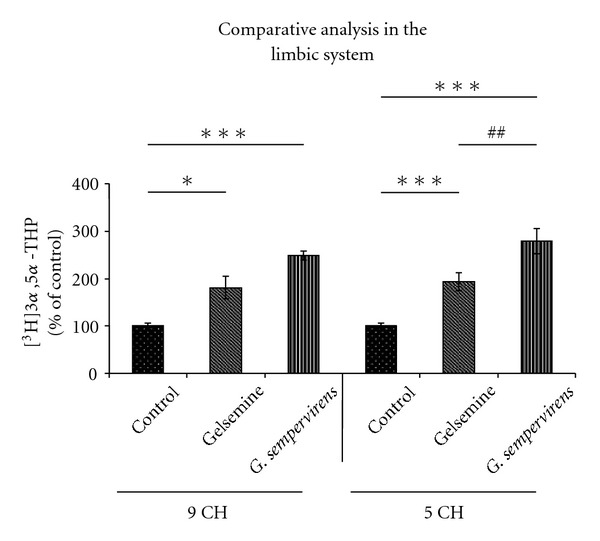
Comparative analysis of the effects of gelsemine and *G. sempervirens* at 9 or 5 cH on [^3^H]PROG conversion into [^3^H]3*α*,5*α*-THP by H-A slices. The values were obtained from experiments similar to those presented in Figure 1. Results were then expressed as percentages of the amount of [^3^H]3*α*,5*α*-THP formed in absence of gelsemine and *G. sempervirens* (control). Each value is the mean (± SEM) of four independent experiments. *P < .05, ***P < .001 as compared to control; ^##^ *P *< .01 as compared to gelsemine (ANOVA followed by Bonferroni's test).

In the limbic system or H-A slices, *G. sempervirens* at 5 cH increased 3*α*,5*α*-THP production 1.5-fold than gelsemine at 5 cH (Figure 5).

### 3.3. Pharmacological Characterization of the Receptor Mediating the Effects of Gelsemine and *G. sempervirens* Preparations on 3*α*,5*α*-THP Biosynthesis in the SC and H-A

**Figure 6:**
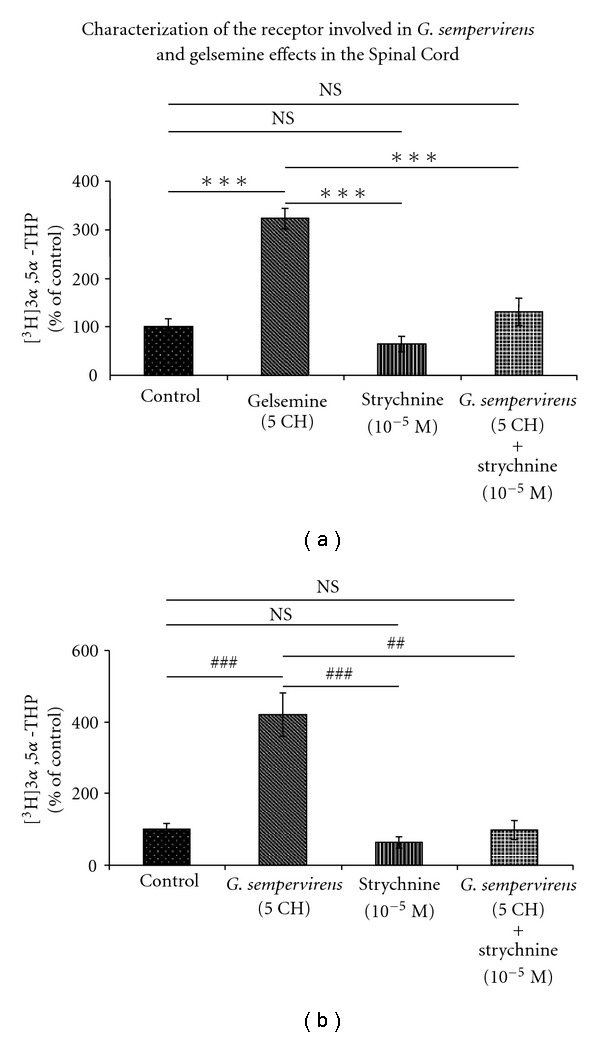
Effects of gelsemine (a) or *G. sempervirens* (b) at 5 cH on [^3^H]3*α*,5*α*-THP production by SC slices in the absence or presence of strychnine (10^−5^ M), the specific glycine receptor antagonist. The values were obtained from experiments similar to those presented in Figure 1. Results were then expressed as percentages of the amount of [^3^H]3*α*,5*α*-THP formed in the control group. Each value is the mean (±SEM) of four independent experiments. ***P < .001 as compared to gelsemine; ^##^ *P* < .01, ^###^ *P* < .001 as compared to *G. sempervirens* (ANOVA followed by Bonferroni's test). NS represents not statistically different.

**Figure 7:**
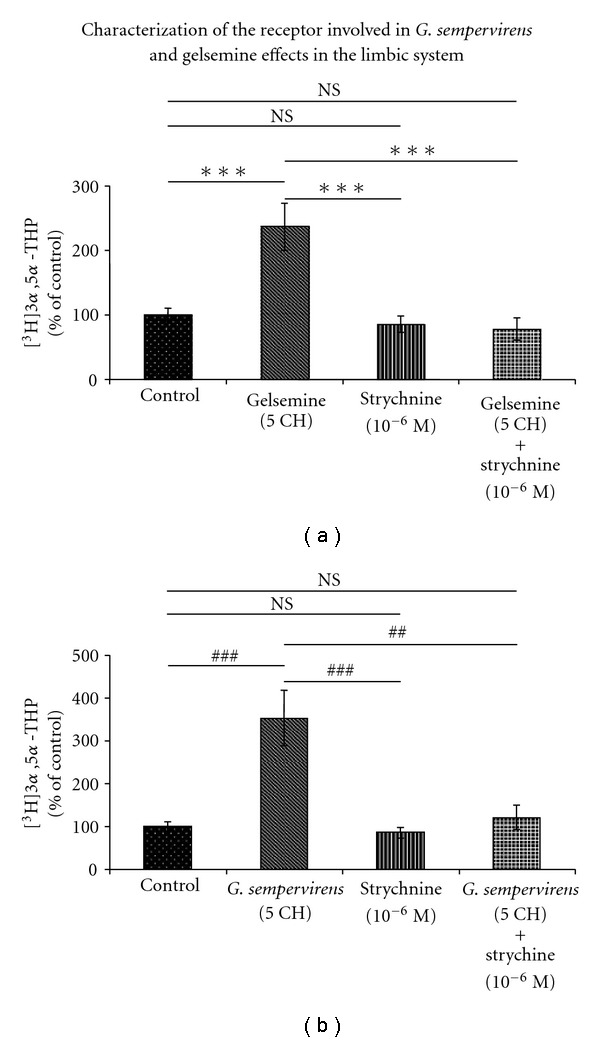
Effects of (a) gelsemine or (b) *G. sempervirens* at 5 cH on 3*α*,5*α*-THP production by H-A slices in the absence or presence of strychnine (10^−6^ M). The values were obtained from experiments similar to those presented in Figure 1. Results were then expressed as percentages of the amount of [^3^H]3*α*,5*α*-THP formed in the control group. Each value is the mean (±SEM) of four independent experiments. ***P < .001 as compared to gelsemine; ^##^ *P* < .01, ^###^ *P* < .001 as compared to *G. sempervirens* (ANOVA followed by Bonferroni's test). NS represents not statistically different.

On the basis of our previous observations about synthetic gelsemine [37], we investigated whether or not strychnine-sensitive Gly-R may be involved in the mediation of the effects of *G. sempervirens* and gelsemine preparations obtained by homeopathic procedure (Figures 6 and 7). In a first step, we performed pulse-chase/HPLC-Flo/One experiments in the presence of various concentrations of strychnine, the well-known selective antagonist of Gly-R; we observed that strychnine at 10^−5^ or 10^−6^ M was completely devoid of action on the formation of [^3^H]3*α*,5*α*-THP in the SC or H-A, respectively (Figures 6 and 7). In addition, we found that the stimulatory effect exerted by gelsemine (5 cH) or *G. sempervirens* (5 cH) on [^3^H]3*α*,5*α*-THP production in the SC was completely antagonized by strychnine at 10^−5^ M (Figures 6(a) and 6(b)). Similarly, in the limbic system or H-A slices, increase of [^3^H]3*α*,5*α*-THP neosynthesis induced by gelsemine (5 cH) or *G. sempervirens* (5 cH) was completely blocked by strychnine (10^−6^ M) (Figures 7(a) and 7(b)).

## 4. Discussion

**Figure 8:**
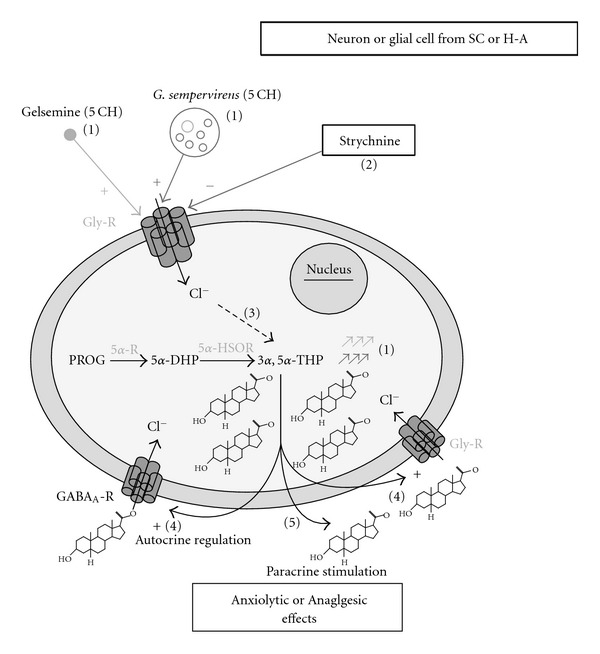
Effect of gelsemine (5 cH) or *G. sempervirens* (5 cH) on 3*α*,5*α*-THP production in the SC and H-A. (1) Gelsemine (5 cH) or *G. sempervirens* (5 cH) stimulated [^3^H]progesterone conversion into [^3^H]3,5-THP in SC and H-A slices. (2) Strychnine, the selective antagonist of glycine receptors (Gly-R), totally blocked the stimulatory effect of gelsemine (5 cH) or *G. sempervirens* (5 cH) on 3*α*,5*α*-THP production. (3) Further investigations are required for the identification of intracellular mechanisms triggered by gelsemine (5 cH) or *G. sempervirens* (5 cH) from Gly-R. However, the stimulatory action exerted by gelsemine (5 cH) or *G. sempervirens* (5 cH) on 3*α*,5*α*-THP production suggests that the intracellular cascade activated by these substances may increase the activity of 3*α*-HSOR which is the key 3*α*,5*α*-THP-synthesizing enzyme. [4, 5] Neurosteroid 3*α*,5*α*-THP newly synthesized by neurons or glial cells in the SC or H-A may modulate GABA_*A*_-R or Gly-R through autocrine (4) or paracrine (5) mechanisms leading to analgesic and/or anxiolytic effects of *G. sempervirens*.

This article provides the first basic evidence supporting the existence of cellular effects of *G. sempervirens* preparations in the limbic system and SC. In addition, the work made possible the identification of pharmacological mechanisms involved in the mediation of *G. sempervirens* and gelsemine action in H-A and SC slices. Thanks to a well-validated approach combining pulse-chase experiments with HPLC analysis and continuous flow scintillation detection [19, 22, 38–42], we observed that gelsemine and *G. sempervirens* at 5 cH significantly stimulated [^3^H]progesterone conversion into [^3^H]3*α*,5*α*-THP in H-A (limbic system) and SC slices. At the dilution 9 cH, a stimulatory action of gelsemine and *G. sempervirens* was also detected on 3*α*,5*α*-THP production in the SC and H-A but the reproducibility rate was limited at 75%. This observation suggests the existence of instability or the lack of homogeneity of high diluted homeopathic solutions. In agreement with this hypothesis, there was no linearity of the stimulatory effects induced by gelsemine or *G. sempervirens* at 5 and 9 cH contrary to what is usually observed in conventional pharmacology for dose-response studies. Neurosteroid 3*α*,5*α*-THP possesses an important therapeutical potential owing to its key role in the regulation of cellular mechanisms involved in anxiety, pain, depression and neurodegeneration [4, 9, 10, 12, 13]. In particular, it has clearly been shown that the endogenous conversion of progesterone into 3*α*,5*α*-THP in the limbic system is crucial for the expression of anxiolytic effect of progesterone [9, 20, 21, 53]. Moreover, we have recently demonstrated that 3*α*,5*α*-THP endogenously synthesized in SC exerts a key analgesic action in animals subjected to sciatic nerve injury-induced neuropathic pain [18, 19]. Therefore, it appears that the stimulatory effect exerted by *G. sempervirens* or gelsemine preparations on 3*α*,5*α*-THP production in H-A or SC may reflect cellular mechanisms activated by these preparations to induce anxiolytic or analgesic effects. In support of this hypothesis, the presence and activity of 3*α*-hydroxysteroid dehydrogenase or 3*α*-HSOR (the key enzyme synthesizing 3*α*,5*α*-THP) has been evidenced in the limbic system and spinal circuit, suggesting that *G. sempervirens* and gelsemine may increase 3*α*,5*α*-THP formation through stimulation of 3*α*-HSOR enzymatic activity in H-A and SC neural networks [18, 54–56]. Furthermore, it has been demonstrated that the limbic and spinal systems, two pivotal structures modulating respectively anxiety and pain sensation, contain several populations of nerve cells expressing Gly-R [43, 44, 46–48, 57–60]. Consistently, our pharmacological analyses revealed that strychnine, the selective antagonist of Gly-R [58, 59], completely blocked the stimulatory effect of gelsemine and *G. sempervirens* on 3*α*,5*α*-THP production in H-A and SC slices. Taken together, these results show that gelsemine (5 cH) and *G. sempervirens* (5 cH), acting through Gly-R located on H-A and SC nerve cells, may stimulate 3*α*-HSOR enzymatic activity, which, in turn, may increase 3*α*,5*α*-THP production in the limbic system and spinal circuit. Our data and hypothesis are summarized and illustrated by the general diagram presented in Figure 8.

The comparative analyses also revealed that the stimulatory capacity of *G. sempervirens* on 3*α*,5*α*-THP biosynthesis in H-A and SC slices seems higher than that of gelsemine. Indeed, the theoretical estimation of gelsemine quantity in *G. sempervirens* mother tincture made after HPLC analysis showed that the tincture contains a concentration less than 1 M. Significant differences are observed between samples of mother tinctures but it appeared that the initial gelsemine concentration in *G. sempervirens* mother tincture may not be higher than 5 × 10^−3^ M. Additional analyses such as mass spectrometry quantification after HPLC or gaz chromatographic purification will certainly help in the future to determine the accurate concentration of gelsemine in *G. sempervirens* mother tincture. However, based on the estimation performed herein, gelsemine at 5 cH (prepared from synthetic gelsemine stock solution at 1 M) may correspond to 10^−10^ M while *G. sempervirens* at 5 cH (prepared from the mother tincture) may contain a concentration of gelsemine within 5 × 10^−14^ M and 5 × 10^−13^ M. Because *G. sempervirens* at 5 cH increased 3*α*,5*α*-THP production 1.5- to 1.7-fold than gelsemine at 5 cH, it is possible to speculate that a positive synergism exists between gelsemine and other constituents present in *G. sempervirens* composition such as sempervirine, gelsemicine and gelsenicine [61–63]. Whether all constituents of *G. sempervirens* also modulate Gly-R like gelsemine [37] remains a matter of speculation. Future investigations will answer this question even though pharmacological analyses described herein revealed that the stimulatory action of *G. sempervirens* (5 cH) was totally (not partially) blocked by strychnine.

At the dilution 9 cH, gelsemine and *G. sempervirens* exerted a stimulatory action on 3*α*,5*α*-THP production but the effect was not reproducible in all intra- and inter-assays performed. However, because the stimulatory effect was observed in 75% of the total number of samples analyzed, it appears that high dilutions of *G. sempervirens* may conserve interesting bioactivity. Therefore, it seems reasonable to expect only a few or no side effects from *G. sempervirens*-based therapeutical strategies. Indeed, even if the results obtained with the dilution 9 cH may not be exploitable because of the limited reproducibility, therapeutic strategies may be achieved with *G. sempervirens* at 5 cH which remains a very low concentration that exhibited a full (100%) reproducible effect on 3*α*,5*α*-THP production in SC and H-A.

In conclusion, the results provided herein may constitute key basic knowledge on cellular and pharmacological effects of *G. sempervirens* and gelsemine preparations. The data may also open new possibilities for the improvement of current therapeutical utilization of *G. sempervirens* which refers only to empirical knowledge but not to fundamental evidence supplied by basic research.

## Figures and Tables

**Figure fig3:**
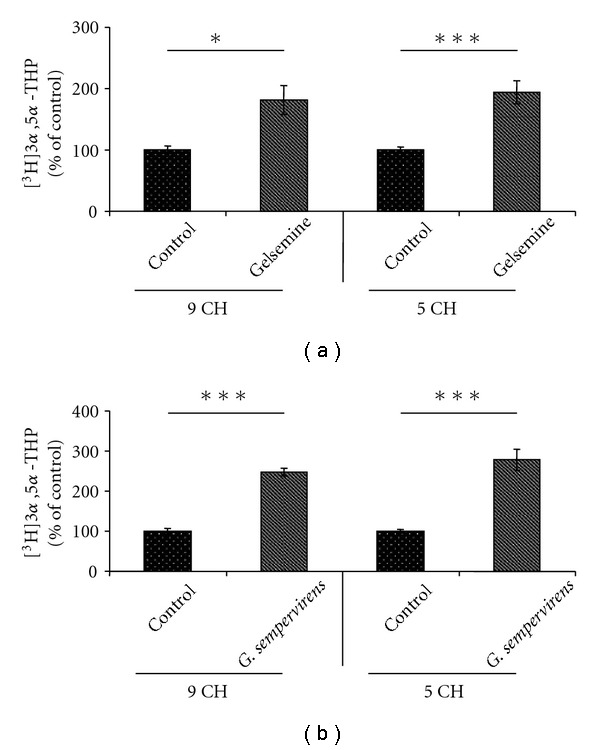
Effects of gelsemine (a) and *G. sempervirens* (b) at 9 or 5 cH on 3*α*,5*α*-THP production by H-A slices. The values were obtained from experiments similar to those presented in Figure 1. Results were then expressed as percentages of the amount of [^3^H]3*α*,5*α*-THP formed in the control group. Each value is the mean (±SEM) of four independent experiments. *P < .05, ***P < .001 as compared to control (Student's *t*-test).
